# Correlation of Single Nucleotide Polymorphisms of *PRM1, PRM2, PYGO2*, and *DAZL* Genes with Male Infertility in North West of Iran

**DOI:** 10.5152/tud.2022.22032

**Published:** 2022-09-01

**Authors:** Elham Ghadirkhomi, Seyed Abdolhamid Angaji, Behnaz Beikzadeh, Mohammad-Reza Mashayekhi, Akram Ghadirkhomi

**Affiliations:** 1 Clinical Research Development Unit of Tabriz Valiasr Hospital, Tabriz University of Medical Sciences, Tabriz, Iran; 2 Department of Cell and Molecular Biology, Kharazmi University Faculty of Biological Sciences, Tehran, Iran; 3 Department of Molecular Genetics, Tarbiat Modares University Faculty of Biological Sciences, Tehran, Iran; 4 Department of Genetics, Tabriz Branch, Islamic Azad University Faculty of Biological Science, Tabriz, Iran; 5 Food and Drug Administrator, University of Medical Sciences, Isfahan, Iran

**Keywords:** Male infertility, DAZL, PYGO2, PRM1, PRM2

## Abstract

**Objective:**

Almost half of infertility is related to male factors. Although the effect of genetic factors on male infertility is identified, about 30%-50% still has no proven cause and is classified as idiopathic infertility. This study was performed to investigate the correlation of some single nucleotide polymorphisms of *PYGO2, DAZL, PRM1*, and *PRM2* genes with male infertility in idiopathic cases among the Iranian population.

**Material and methods:**

In this case-control study, 120 idiopathic azoospermia or severe oligospermia patients in the range of 25-45 years and 120 fertile men in the same age range were recruited as case and control groups, respectively. Eight different single nucleotide polymorphisms including *PRM1 rs737008*, *PRM1 rs423668, PRM2 rs1646022, PRM2 rs11645592, PYGO2 rs141722381, PYGO2rs61758741, DAZL rs75931701*, and *DAZL rs188506466* were genotyped by using amplification-refractory mutation system polymerase chain reaction methods. Hardy-Weinberg was calculated by using online website. Statistical Package for Social Sciences software was applied for statistical analysis. *P* value <.05 was considered significant. Thirty percent of the samples were regenotyped to confirm the obtained results.

**Results:**

The obtained results showed a significant correlation between *PYGO2 rs141722381* in the heterozygote form (odds ratio: 2.803, 95% CI: 1.397-5.626). Heterozygote over-dominance was also observed in this variant (odds ratio: 2.637, 95%CI: 1.321-5.264). There was no significant association between other studied single nucleotide polymorphisms and male infertility.

**Conclusion:**

This study proposed a novel single nucleotide polymorphism as a predisposition of male infertility among the Iranian population, but more studies in larger populations are needed to confirm the results.

**Main Points**

- About 15% of couples have infertility, a health problem that is increasing globally.
- Male factors contribute 40%-60% of cases of infertility. Genetic factors are reported in about 4% of infertile men, but the vast majority of infertility in men (50%-60%) have no distinct causes yet and are classified as idiopathic infertility.
- Genetic diversity involved in spermatogenesis process may play an important role in sperm production defects and male infertility.
- Investigating the association of single nucleotide polymorphisms of the key genes involved in this process is one of the most exciting areas of male infertility research worldwide.
- The obtained results can be used by urologists in infertility treatment centers as a predisposition for male infertility in idiopathic cases.

## Introduction

Male infertility affects about 7% of the male population, a figure that is increasing.^1^ Male infertility is usually associated with decreased sperm count.^2^ The role of genetic factors in male infertility is well-known, such as Klinefelter syndrome, Y-chromosome microdeletions, and cystic fibrosis. Genetic factors are reported in about 4% of infertile men, but the vast majority of infertility in men (50%-60%) have no distinct causes yet and are classified as idiopathic infertility.^1^ The sperm production process which is called spermatogenesis is a complex process involving spermatogonia stem cell proliferation, meiosis, and spermatid differentiation. Genetic diversity involved in this process may play an important role in spermatogenesis defects and male infertility.^3,4^ *Pygo2, DAZL*, and *PRM1* are some genes involved in spermatogenesis which are reported to play the main role in male infertility.^5^ Human *PYGO2* protein is localized in the nucleus and composed of 2 domains: the zing finger motif (PHD) and the N-terminal homology domain (NHD).^6^ *PYGO2* acts as a co-activator of the Wnt pathway. N-terminal homology domain of this gene, with the help of beta-catenin, BCL9, and LEF/TCF transcription factors, forms the transcription complex and leads to the expression of some specific target genes.^7^ N-terminal homology domain also plays an important role in histone methylation.^8^ *Pygo2* gene is located on chromosome 1q21.3 and is expressed during sperm chromatin remodeling.^9^ The effects of *pygo2* targeted mutations in mice have shown that the limited activity of *pygo2* during spermatogenesis leads to a selective decrease in the expression of essential post-meiotic genes for chromatin condensation including protamine (P1 and P2), transfer protein 2 (*TNP2*), and H1fnt genes which may lead to infertility.^10^ It has also been shown that decreased pygo2 activity in elongated spermatids leads to disruption of histone H3 acetylation suggesting that pygo2 recruit HAT facilitates H3 acetylation and further histone-to-protamine transitions.^11,12^

DNA in the sperm head is highly compacted, which is vital to maintain the hydrodynamic shape of the sperm and the genomics integrity.^13^ At the last stage of the spermatogenesis process, histones are replaced with protamines to provide this level of chromatin condensation.^14^ There are 2 types of protamines (*PRM1* and *PRM2*) in humans that are located on chromosome 16.^15^ PRMs are highly basic and rich in arginine residue, which provides strong bindings to negatively charged DNA.^16^ More than 20 single nucleotide polymorphisms (SNPs) are reported in *PRM1* and *PRM2* genes.^17^ Various studies have reported an association between *PRM2* and male infertility.^16^ Jiang et al^18^ released a report indicating *PRM2* rs1646022 polymorphisms and the risk of male infertility. Iguchi et al^19^ have reported a correlation of *PRM1* c.197G>T polymorphism and male infertility. It was also suggested that *PRM1* c.-190C>A polymorphism may lead to male infertility due to abnormal sperm morphology.^20^

Another gene that plays an important role in spermatogenesis is *DAZL*. *DAZL* is expressed in primordial germ cells (PGCs)^21^ and produces an RNA-binding protein that controls the growth, differentiation, and maturation of germ cells.^22^ DAZL protein acts as an activator of transcription and is present in the nucleus of gonocytes and spermatogonia.^23^ Lin et al^23^ reported that *dazl *transcripts were lower in the testes of azoospermia cases than in fertile men. Also, it is suggested that approximately 10% of patients with azoospermia and oligospermia show deletion in the DAZ family gene.^24^

In this study, we tried to find the association of some SNPs of *PYGO2*,* PRM1*,* PRM2*, and *DAZL* genes with male infertility. Single nucleotide polymorphisms were selected for the study which lead to a stop codon or missenses that alter the nature of the amino acids which in turn can produce truncated proteins or proteins with improper function.

## Materials and Methods

In this case–control study, the association of the studied SNPs and their majority or minority effect on male infertility was investigated to identify biomarkers that can be used for diagnosis and prognosis of male infertility in medical centers.

**Participants:** A total of 120 case samples were selected among infertile men referred to the assisted reproductive technology center of the Valiasr Hospital (Tabriz, Iran) between 2019 and 2020. Control subjects were 120 fertile healthy men with normal spermogram who had at least 1 child at the time of study. All the participants were in the range of 30-45 years old. Case samples were idiopathic azoospermia or oligospermia. To exclude samples that have a possible cause, all cases were subjected to physical examination, and necessary hormonal (Follicle-stimulating hormone (FSH), Thyroid stimulating hormone (TSH), luteinizing hormone (LH), testosterone, and prolactin) and genetic tests (karyotyping and microdeletions of Y chromosome, *CFTR*, etc.) were taken. Semen analysis was performed twice for all participants using microscopic methods and according to World Health Organization criteria (WHO, 2010).^25^ The sperm count of infertile patients was <5×10^6^. All the parameters for control subjects were in the normal range.

Sample size influences random sampling error. In order to control type I and type II errors, the sample size was obtained using the statistical sample size calculation formula.^26^ The ratio of the case to control (r) was considered 1. The *P* value was considered similar to the standard deviation (0.29). The probability of exposure in case (P2) and control groups (P1) was 0.38 and 0.2, respectively. The minimum sample size was 100 individuals, but more (120) were recruited to increase power calculation.

### Ethical Considerations

This study has been carried out in compliance with all the rules and instructions related to medical research in Iran. Written consent was obtained from all participants in this project. The project was approved by the Science and Research Branch of Tehran’s Islamic Azad University Ethical Committee (IR.IAU.SRB.REC.1398.001). STORBE guidelines were followed in this study.

**DNA Extraction**: 3 mL of venous blood was collected from all participants in the ethylenediaminetetraacetic acid (EDTA) containing venoject tubes. DNA was extracted using PCR Bio Rapid Extract kit (PCR Biosystems, London, N6 4ER, UK). The yield and purity of DNA samples (OD 260/280) were measured by using a nanodrop (Denovix Ds-11 spectrophotometer).

**Genotyping:** Amplification refractory mutation system method was used for genotyping the studied polymorphisms. Primers were designed by Primer 3 software. Oligoanalyzer and primer blast softwares were used to check the quality and specificity of the primers. Polymerase chain reaction products were visualized on 2% agarose gel electrophoresis by using Novel juice stain (Cat.No.LD001-1000). The obtained results were confirmed by regenotyping 30% of samples.

**Primers and Cycling Condition:** Polymerase chain reaction was performed in a final volume of 25 µL. Ready to use mastermix (PCR Biosystems Ltd) was used to amplify the target gene. Cycling condition for all the reactions was 95°C for 5 minutes for the first cycle, followed by denaturation at 94°C for 45 seconds, annealing temperature according to optimum Tm of each reaction (Table 1) for 40 seconds, extension at 72°C for 40 seconds for 29-30 cycle and then final extension at 72°C for 7 minutes. The sequence of primers is summarized in Table 1.

### Statistical Analysis

In order to find a significant difference between the case and control groups in relation to each of the studied SNPs, minor allele frequencies (MAFs) were estimated by using excel software and compared between the 2 groups.

To determine the statistical analysis pattern, Hardy Weinberg’s equilibrium (HWE) and chi-square were calculated. Obtained χ^2^ was compared with standard statistical table (3.8). If it was smaller than 3.8, the group is in HWE otherwise HWE is disturbed in that group.^27^ The status of HWEin the case and control group was obtained using online software (https://wpcalc.com/en/equilibrium-hardy-weinberg/). If both the case and control groups were in HWE, the multiplicative model was used for statistical analysis. If the control group was under HWE but the case group was not, dominant/recessive model was used; otherwise, additive model was performed. The association of genotypes with male infertility was analyzed by calculating odds ratios (ORs) with 95% CIs via logistic regression. Statistical analysis was performed IBM SPSS Statistics v.22 (IBM SPSS Corp.; Armonk, NY, USA). *P *< .05 was considered significant.

## Results

Semen analysis parameters are summarized in Table 2. The concentration of extracted DNA was in the range of 77-297 ng/µL and obtained OD 260/280 was in the range of 1.79-1.9. The frequency of the studied genotypes and their association with male infertility are summarized in Table 3. There was an association between *PYGO2 *rs141722381 and male infertility, but no relationship was found between the other studied SNPs and male infertility.

The obtained *P* values comparing MAF between case and control groups showed a significant difference between MAF of infertile patients and controls in the case of *PYGO2 rs141722381* (*P* value = .007), but there was no such a difference between case and control groups in the other studied SNPs. The results are summarized in Table 3.

In all studied genotypes, obtained χ^2^ indicated neither the case group nor the control group was in HWE (Table 4). Since the additive model is independent of HWE and our samples showed an imbalance, the additive model was used for association analysis. There was a significant association between *PYGO2141722381* and male infertility in the heterozygote form (OR: 2.803, CI: 1.397-5.626). Heterozygote over-dominance was also observed in this polymorphism (OR: 2.637, CI: 1.321-5.264). No significant association was found between the other studied polymorphism and male infertility. The association of each genotype with male infertility is summarized in Table 5. Heterozygote over-dominance was not observed in other studied SNPs (Table 5).

## Discussion

Spermatogenesis is a complex process of mitosis and meiosis in tandem to produce haploid sperm, in which at least 150 different genes play a key role and any defect of these genes may affect the production of healthy and functional sperm.^18^ Since almost half of the male infertility has no definite cause and is classified as idiopathic infertility, analysis of the possible association of genes involved in the spermatogenesis process with male infertility remains an area of active investigation.^28^ In this study, we investigated the correlation of 6 different SNPs involved in spermatogenesis with male infertility in idiopathic cases, which was not studied before.

Chromatin condensation during sperm remodeling is one of the important procedures through spermatogenesis.^17^ Histones are first replaced with TNP1 and TNP2 proteins, and then these proteins are replaced with PRM1 and PRM2 proteins. This causes maximum compression of the chromatin to be placed in the sperm head.^15^ *Pygo2* gene is also essential for chromatin remodeling and regulate *TNP2*and *PRM* gene expression via histone methylation changes.^11^ In the present research, there was no association between studied SNPs of *PRM* genes* (PRM2* rs1646022, *PRM2*rs11645592, *PRM1*rs737008, and *PRM1*rs423668) and male infertility among the Iranian population. But results revealed a critical correlation between *PYGO2*rs141722381 in the heterozygote form (OR: 2.803, 95%CI: 1.397-5.626) and male infertility. There was also no association between *PYGO2*61758741 and male infertility. *Pygo2* rs141722381 leads to damage to the tertiary structure of protein and rs61758741 is a missense mutation that replaces a basic amino acid with an acidic amino acid.^29^ It is reported that 2 SNP mutations in *PYGO2* (rs61758740 and rs141722381) are correlated to idiopathic azoospermia.^6^ Moud et al^5^ have also reported that there is a relationship between *PYGO2 *rs61758740 and male infertility among the Iranian population, but there is no significant correlation between rs61758741 and male infertility^5^ which confirms our findings.

Two separate studies among the Spanish and Japanese populations showed an association between the *PRM1* gene (c.-190C>A) and male infertility.^17,20^ A meta-analysis study that examined the correlation of 6 different SNPs (rs35576928, rs737008, rs35262993, rs2301365, rs1646022, and rs2070923) with male infertility has also defined *PRM1* c.-190C>A polymorphism (rs2301365) as a risk factor of male infertility, but no association is reported between the other studied SNPs and male sterility^18^ which is consistent with our results. The *PRM2* c.248C>T (rs74007626) polymorphism that replaces glutamine amino acid codon with stop-codon is also reported benign among the Japanese and Iranian populations.^17^ In contrast with our findings, a meta-analysis study has reported a correlation between *PRM2* rs1646022 polymorphism and an increased risk of male infertility.^15^ The difference in ethnicity, control source, sample size, and genotyping method may be the cause of contradictory results. Publication year is also reported as a confounding factor in the association between *PRM2* rs1646022 polymorphism and male infertility.^15^ As far as we know, it was the first study investigating the association of rs423668 and rs11645592 with male infertility.

Microdeletions of *DAZ* gene family on the chromosome Y are one of the well-characterized causes of infertility in azoospermia and oligospermia patients.^30^ *DAZ-like (DAZL*), an autosomal homolog of *DAZ*, is expressed in germ cells and is essential for spermatogenesis.^30^ According to the meta-analysis review in 2016, there is no correlation between SNP260 (rs121918346) of *DAZL* and azoospermia while SNP386 (rs121918346) of this gene is related to male infertility only among the Chinese Han population.^30^ A significant association is also reported between T54A polymorphism and male infertility in only Asian population.^31^ An important SNP N109T (rs75931701) in the coding sequence of the gene was predicted to be deleterious by computational methods which may affect the structure and/or function of DAZL significantly,^24^ but contrary to expectations in the present study, no significant difference was observed among the allele frequency of case and control group. There was also no association between *DAZL*rs188506466 and male infertility among the Iranian population. As of our knowledge, this was also the first study that investigated the effect of this SNP on male infertility. Confounders such as smoking, alcohol consumption, some environmental factors, high-risk jobs, and so on were not included in the study which was the limitation of this study.

In conclusion, the results of the study revealed an important association between *PYGO2*rs141722381 and male infertility that suggests a novel risk factor for idiopathic cases. The obtained results can be used by urologists in infertility treatment centers as a predisposition of male infertility in idiopathic cases, but the results should be validated by different populations first.

## Figures and Tables

**Table 1.** Sequence of Primers and Annealing Temperatures

Primer Type	Primer Sequence	Annealing Tm (°C)	Product Size (bp)
PRM1 rs737008G>A		55.5°C for 45 seconds	189
Wild type forward primer	5´-AACGCTGTCACCATTGTCG-3´		
Mutant type forward primer	5´-GAACGCTGTCACCATTGTCA-3**´**		
Common reverse primer	5´-TCTTCTTGGTGCATTGTGG-3´
PRM1 rs423668 C>T		58.5°C for 45 seconds	193
Wild type forward primer	5´- TTAGCCAGGTGTGGTGGC-3´		
Mutant type forward primer	5´- TTAGCCAGGTGTGGTGGT-3´		
Common reverse primer	5´- ACAGTTGTCACGCTGGGTTT-3´
PRM2 rs1646022 C˃G		55°C for 30 seconds	171
Wild type forward primer	5’- GGGTGGTCAGGGACATAC- 3’		
Mutant type forward primer	5’- GGGTGGTCAGGGACATAG-3’		
Common reverse primer	5’- GGCCAGTCTCACTATAGGC-3’
PRM2 rs11645592 G>A		56°C for 30 seconds	
Wild type reverse primer	5´-GGAGCAGATGACTATTTTGGAG-3’		
Mutant type reverse primer	5´-GGAGCAGATGACTATTTTGGAA-3’		
Common forward primer	5´- CATTCCTCACTCTTACAATCCTC-3’
PYGO2 rs141722381 T>A		56°C for 40 seconds	233
Wild type forward primer	5´- CCAGGAAAGGGACTTGTGT-3´		
Mutant type forward primer	5´- CCAGGAAAGGGACTTGTGA-3´		
Common reverse primer	5´- CAGCCTCTGGGTCAAAACTT-3´
PYGO2 rs61758741 T>C		57°C for 40 seconds	171
Wild type forward primer	5´- CAG GTG GAT TCA AGG GCT T- 3´		
Mutant type forward primer	5´- CAG GTG GAT TCA AGG GCT C-3´		
Common reverse primer	5´- CTC TGT CCC AAC GAT TTG CT-3´
DAZL rs75931701 T>G		55°C for 35 seconds	218
Wild type forward primer	5´- ACGTGGCTAGAGTTCAGAT-3´		
Mutant type forward primer	5´-ACGTGGCTAGAGTTCAGAG-3´		
Common reverse primer	5´-TTGTCACATCATCGAACCTT-3´
DAZL rs188506466 G>A		59.5°C for 45 seconds	549
Wild type forward primer	5´-TGTGGGCCATTTCCAGAGGG-3´		
Mutant type forward primer	5´-TGTGGGCCATTTCCAGAGGA-3´		
Common reverse primer	5´-CTCTGCCTCTGGCTTTACCA-3´

**Table 2.** Semen Characteristic

Semen Characteristic	Fertile Controls (n = 120)	Infertile Cases (n = 120)	Lower Reference Limit (WHO 2010)
Volume, ml	2-8.5	1-8	1.5
Total sperm count in ejaculate (×10^6^/mL)	50-120	<10 39	
Sperm count ×10^6^/mL	35-95	0-5	15
Total motility, %	45-85	–	42(38-42)
Progressive motility (PR), %	42-80	–	32(31-34)
Vitality, %	55-65	–	58 (55-63)
Normal morphology %	4-5	0-1	3-4
PH	7.2-8	7.2-7.6	>7.2
Viscosity	N	N- M-H^*^N	Smooth & watery
Liquefaction time	20-35 min	20-45 min	<60 min
Round cells ×10^6^/mL	0-4	0-4	<5
Sperm agglutination	0-1	0-1	<2

*N, Normal; M, Moderate; H, High.

**Table 3.** Allele Frequency and Distribution of the *PRM2*, *PYGO2*, and *DAZL* Genotypes in Infertile Patients and Controls

Genotype	Cases (n = 120) (%)	Controls (n = 120)	MAF^*^ (Case Group) %	MAF (Control Group) %	*P* ^*^
*PRM2 *1646022 CC	12 (10.0)	20 (16.7)	55	50	.292
*PRM2 *1646022 CG	84 (70.0)	80 (66.7)			
*PRM2 *1646022 GG	24 (20)	20 (16.7)			
*PRM2 *11645592 GG	57 (47.5)	68 (56.7)	36.25	29.16	.338
*PRM2 *11645592 GA	39 (32.5)	34 (28.3)			
*PRM2 *11645592 AA	24 (20)	18 (15.0)			
*PRM1 *737008GG	79 (65.8)	80 (66.7)	24.58	25.41	.734
*PRM1 *737008GA	23 (19.2)	19 (15.8)			
*PRM1 *737008AA	18 (15.0)	21 (17.5)			
*PRM1 *423668 CC	52 (43.3)	56 (46.7)	43.75	38.33	.413
*PRM1 *423668 CT	31(25.8)	36 (30.0)			
*PRM1 *423668 TT	37 (30.8)	28 (23.3)			
*PYGO2* 61758741 TT	7 (5.8)	10 (8.3)	52.5	48.75	.305
*PYGO2* 61758741 TG	100 (83.3)	103 (85.8)			
*PYGO2 *61758741GG	13 (10.8)	7 (5.8)			
*PYGO2 *141722381TT	79 (65.8)	100 (83.3)	21.25	10.83	.007
*PYGO2 *141722381TA	31 (25.8)	14 (11.7)			
*PYGO2 *141722381AA	10 (8.3)	6 (5)			
*DAZL *75931701 TT	89 (74.2)	98 (81.7)	17.5	12.5	.374
*DAZL *75931701 TG	20 (16.7)	14 (11.7)			
*DAZL *75931701 GG	11 (9.2)	8 (6.7)			
*DAZL *188506466 GG	45 (37.5)	53 (44.2)	46.25	39.16	.377
*DAZL *188506466 GA	39 (32.5)	40 (33.3)			
*DAZL *188506466 AA	36 (30.0)	27 (22.5)			

*P* values were obtained from χ^2^ test.

*MAF, minor allele frequency in studied case and control group.

**Table 4.** Hardy-Weinberg Equilibrium (HWE) Status in Case and Control Group

Genotype	χ^2^ for Case	χ^2^ for Control
*PRM2*rs1646022	20.5816	13.3333
*PRM2* rs11645592	10.5724	11.8531
*PRM1* rs737008	28.006	40.6999
*PRM1* rs423668	27.0901	16.0267
*PYGO2*rs6175874	54.0038	61.8181
*PYGO2* rs141722381	6.2455	18.8291
*DAZL* rs75931701	21.4511	26.1333
*DAZL* rs188506466	14.3938	10.8357

χ^2^, Chi-squared. In order to analyze HWE status in each group, calculated χ^2^ was compared with standard statistical table (3.8). If it was smaller than 3.8, the group is in HWE otherwise HWE is disturbed in that group.

**Table 5.** Genetic Models of Single Nucleotide Polymorphism Associated with Male Infertility

Genotype	OR (95%CI)	*P *
*PRM2* 1646022 CC	1.00	
*PRM2* 1646022 GC/CC	1.750 (0.803-3.812)	.156
*PRM2* 1646022 GG/CC	2.000 (0.789-5.067)	.142
*PRM2* 1646022 GC/CC+GG	1.167 (0.677- 2.011)	.579
*PRM2* 11645592 GG	1.00	
*PRM2* 11645592 GA/GG	1.368 (0.767-2.442)	.288
*PRM2* 11645592 AA/GG	1.591 (0.786-3.220)	.195
*PRM2* 11645592 GA/AA+GG	1.218 (0.702-2.113)	.483
*PRM1* 117752382 AA	1.00	
*PRM1* 117752382 AT/AA	1.226 (0.619-2.426)	.558
*PRM1* 117752382 TT/AA	0.868 (0.430-1.752)	.693
*PRM1* 117752382 AT/AA+TT	1.260 (0.646-2.459)	.497
*PRM1* 423668 CC	1.00	
*PRM1* 423668 CT/CC	0.927 (0.503-1.708)	.809
*PRM1* 423668 TT/CC	1.423 (0.766-2.643)	.263
*PRM1* 423668 CT/CC+TT	0.813 (0.462-1.430)	.472
*PYGO2*61758741 TT	1.00	
*PYGO2*61758741GT/TT	1.387 (0.508-3.786)	.522
*PYGO2*61758741GG/TT	2.653 (0.699-10.063)	.147
*PYGO2*61758741GT/TT+GG	0.825 (0.409-1.666)	.592
*PYGO2* 141722381 TT	1.00	
*PYGO2*141722381 AT/TT	2.803 (1.397-5.626)	.003
*PYGO2*141722381AA/TT	2.110 (0.735-6.054)	.158
*PYGO2*141722381AT/TT+AA	2.637 (1.321-5.264)	.005
*DAZL* 75931701 TT	1.00	
*DAZL* 75931701 GT/TT	1.573 (0.750-3.300)	.228
*DAZL* 75931701 GG/TT	1.514 (0.583-3.934)	.392
*DAZL* 75931701 GT/TT+GG	1.514 (0.726-3.160)	.267
*DAZL* 188506466 GG	1.00	
*DAZL* 188506466 GA/GG	1.148 (0.634-2.079)	.648
*DAZL* 188506466 AA/GG	1.570 (0.830-2.972)	.164
*DAZL* 188506466 GA/GG+AA	0.963 (0.562-1.650)	.891

## References

[1] Houston BJ Riera-Escamilla A Wyrwoll MJ et al. A systematic review of the validated monogenic causes of human male infertility: 2020 update and a discussion of emerging gene–disease relationships. Hum Reprod Update. 2021;28(1):15 29. 10.1093/humupd/dmab030) 34498060 PMC8730311

[2] Wang W Lu N Xia Y et al. FAS and FASLG polymorphisms and susceptibility to idiopathic azoospermia or severe oligozoospermia. Reprod Biomed Online. 2009;18(1):141 147. 10.1016/s1472-6483(10)60436-1) 19146781

[3] Ying HQ Scott MB Zhou-Cun A . Relationship of SNP of H2BFWT gene to male infertility in a Chinese population with idiopathic spermatogenesis impairment. Biomarkers. 2012;17(5):402 406. 10.3109/1354750X.2012.677066) 22509975

[4] Ghadirkhomi E Angaji SA Khosravi M Mashayekhi MR . Association of novel single nucleotide polymorphisms of genes involved in cell functions with male infertility: a study of male cases in Northwest Iran. J Reprod Infertil. 2021;22(4):258-266. 10.18502/jri.v22i4.7651) PMC8669412 34987987

[5] Moud SS Seraji KK Ramezani M Piravar Z . Associations of PYGO2 and PRDM9 Genes Single Nucleotide Polymorphisms with Idiopathic Azoospermia in Iranian Population; 2021. 10.30476/IJMS.2022.93009.2433 PMC9843457 36688188

[6] Ge SQ Grifin J Liu LH et al. Associations of single nucleotide polymorphisms in the Pygo2 coding sequence with idiopathic oligospermia and azoospermia. Genet Mol Res. 2015;14(3):9053 9061. 10.4238/2015.August.7.14) 26345837

[7] Gu B Sun P Yuan Y et al. Pygo2 expands mammary progenitor cells by facilitating histone H3 K4 methylation. J Cell Biol. 2009;185(5):811 826. 10.1083/jcb.200810133) 19487454 PMC2711593

[8] Cantù C Valenta T Hausmann G Vilain N Aguet M Basler K . The Pygo2-H3K4me2/3 interaction is dispensable for mouse development and Wnt signaling-dependent transcription. Development. 2013;140(11):2377 2386. 10.1242/dev.093591) 23637336

[9] Nair M Nagamori I Sun P et al. Nuclear regulator Pygo2 controls spermiogenesis and histone H3 acetylation. Dev Biol. 2008;320(2):446 455. 10.1016/j.ydbio.2008.05.553) 18614164 PMC2553271

[10] Bettegowda A Wilkinson MF . Transcription and post-transcriptional regulation of spermatogenesis. Philos Trans R Soc Lond B Biol Sci. 2010;365(1546):1637 1651. 10.1098/rstb.2009.0196) 20403875 PMC2871917

[11] Wang T Gao H Li W Liu C . Essential role of histone replacement and modifications in male fertility. Front Genet. 2019;10:962. 10.3389/fgene.2019.00962) PMC6792021 31649732

[12] Wang X Kang JY Wei L et al. PHF7 is a novel histone H2A E3 ligase prior to histone-to-protamine exchange during spermiogenesis. Development. 2019;146(13):dev175547. 10.1242/dev.175547) 31189663

[13] Bisht S Mathur P Dada R . Protamines and their role in pathogenesis of male infertility. Transl Cancer Res. 2016;5(3):324 326. 10.21037/tcr.2016.06.24)

[14] Dehghanpour F Fesahat F Miresmaeili SM Zare Mehrjardi EZ Honarju A Talebi AR . Analysis of PRM1 and PRM2 polymorphisms in Iranian infertile men with idiopathic teratozoospermia. Int J Fertil Steril. 2019;13(1):77-82. 10.22074/ijfs.2019.5650) PMC6334022 30644249

[15] Nemati H Sadeghi M Nazeri M Mohammadi M . Evaluation of the association between polymorphisms of PRM1 and PRM2 and the risk of male infertility: a systematic review, meta-analysis, and meta-regression. Sci Rep. 2020;10(1):17228. 10.1038/s41598-020-74233-3) 33057064 PMC7560625

[16] Hamad MF Quantification of histones and protamines mRNA transcripts in sperms of infertile couples and their impact on sperm’s quality and chromatin integrity. Reprod Biol. 2019;19(1):6 13. 10.1016/j.repbio.2019.03.001) 30876814

[17] Aydos OSE Hekmatshoar Y Altınok B et al. Genetic polymorphisms in PRM1, PRM2, and YBX2 genes are associated with male factor infertility. Genet Test Mol Biomarkers. 2018;22(1):55 61. 10.1089/gtmb.2017.0040) 29227750

[18] Jiang W Sun H Zhang J et al. Polymorphisms in protamine 1 and protamine 2 predict the risk of male infertility: a meta-analysis. Sci Rep. 2015;5(1):15300. 10.1038/srep15300) 26472740 PMC4607923

[19] Iguchi N Yang S Lamb DJ Hecht NB . An SNP in protamine 1: a possible genetic cause of male infertility? J Med Genet. 2006;43(4):382 384. 10.1136/jmg.2005.037168) 16199539 PMC2563215

[20] Yu QF Yang XX Li FX Ye LW Wu YS Mao XM . Association of PRM1-190C-> A polymorphism with teratozoospermia. Zhonghua Nan Ke Xue. 2012;18(4):314 317. 22574365

[21] Alzoubi KH Khabour OF Tashtoush NH Al‐azzam SI Mhaidat NM . Evaluation of the effect of pentoxifylline on sleep‐deprivation induced memory impairment. Hippocampus. 2013;23(9):812 819. 10.1002/hipo.22135) 23592546

[22] Fu XF Cheng SF Wang LQ Yin S De Felici M Shen W . DAZ family proteins, key players for germ cell development. Int J Biol Sci. 2015;11(10):1226-1235. 10.7150/ijbs.11536) PMC4551758 26327816

[23] Lin YM Chen CW Sun HS et al. Expression patterns and transcript concentrations of the autosomal DAZL gene in testes of azoospermic men. Mol Hum Reprod. 2001;7(11):1015 1022. 10.1093/molehr/7.11.1015) 11675467

[24] Nailwal M Chauhan JB . In silico analysis of non-synonymous single nucleotide polymorphisms in human DAZL gene associated with male infertility. Syst Biol Reprod Med. 2017;63(4):248 258. 10.1080/19396368.2017.1305466) 28388287

[25] Tournaye H Krausz C Oates RD . Novel concepts in the aetiology of male reproductive impairment. Lancet Diabetes Endocrinol. 2017;5(7):544 553. 10.1016/S2213-8587(16)30040-7) 27395771

[26] Charan J Biswas T . How to calculate sample size for different study designs in medical research? Indian J Psychol Med. 2013;35(2):121 126. 10.4103/0253-7176.116232) 24049221 PMC3775042

[27] Lewis CM Genetic association studies: design, analysis and interpretation. Brief Bioinform. 2002;3(2):146 153. 10.1093/bib/3.2.146) 12139434

[28] da Silva Costa CS Assessment of Fertility Associated Variants in a Portuguese Cohort of Azoospermia and Severe Oligozoospermia; 2020.

[29] Ge SQ Jeanine G Liu LH et al. Association of single nucleotide polymorphisms (SNPs) in Pygo2 coding gene with idiopathic oligospermia and azoospermia. Yi Chuan= Hereditas. Yi Chuan. 2013;35(5):616 622. 10.3724/sp.j.1005.2013.00616) 23732668

[30] Chen P Wang X Xu C et al. Association of polymorphisms of A260G and A386G in DAZL gene with male infertility: a meta-analysis and systemic review. Asian J Androl. 2016;18(1):96-101. 10.4103/1008-682X.153542) PMC4736364 25994644

[31] Zhang S Tang Q Wu W et al. Association between DAZL polymorphisms and susceptibility to male infertility: systematic review with meta-analysis and trial sequential analysis. Sci Rep. 2014;4(1):4642. 10.1038/srep04642) 24717865 PMC5380160

